# A novel application of entropy analysis for assessing changes in movement variability during cumulative tackles in young elite rugby league players

**DOI:** 10.5114/biolsport.2023.112965

**Published:** 2022-02-18

**Authors:** Bruno Fernández-Valdés, Ben Jones, Sharief Hendricks, Dan Weaving, Carlos Ramirez-Lopez, Sarah Whitehead, Jacob González, Jose Gisbert-Orozco, Michela Trabucchi, Gerard Moras

**Affiliations:** 1 School of Health Sciences, TecnoCampus, Pompeu Fabra University, Spain; 2 National Institute of Physical Education of Catalonia (INEFC), Barcelona, Spain; 3 Unió Esportiva Santboiana, DH Rugby, Sant Boi de Llobregat, Barcelona, Spain; 4 Carnegie Applied Rugby Research (CARR) centre, Institute for Sport, Physical Activity and Leisure, Leeds Beckett University, Leeds, UK; 5 Leeds Rhinos RLFC, Leeds, UK; 6 England Performance Unit, The Rugby Football League, Leeds, UK; 7 School of Science and Technology, University of New England, Armidale, NSW, Australia; 8 Division of Exercise Science and Sports Medicine, Department of Human Biology, Faculty of Health Sciences, the University of Cape Town and the Sports Science Institute of South Africa, Cape Town, South Africa; 9 Yorkshire Carnegie RUFC, Leeds, UK; 10 FC Barcelona, Barcelona, Spain; 11 Department of Civil and Environmental Engineering, Universitat Politècnica de Catalunya (UPC), Barcelona, Spain

**Keywords:** Dynamical systems, Motor Control, Team Sport, Technology, Measurement

## Abstract

The aim of this study was to identify between-position (forwards vs. backs) differences in movement variability in cumulative tackle events training during both attacking and defensive roles. Eleven elite adolescent male rugby league players volunteered to participate in this study (mean ± SD, age; 18.5 ± 0.5 years, height; 179.5 ± 5.0 cm, body mass; 88.3 ± 13.0 kg). Participants performed a drill encompassing four blocks of six tackling (i.e. tackling an opponent) and six tackled (i.e. being tackled by an opponent while carrying a ball) events (i.e. 48 total tackles) while wearing a micro-technological inertial measurement unit (WIMU, Realtrack Systems, Spain). The acceleration data were used to calculate sample entropy (SampEn) to analyse the movement variability during tackles performance. In tackling actions SampEn showed significant between-position differences in block 1 (p = 0.0001) and block 2 (p = 0.0003). Significant between-block differences were observed in backs (block 1 vs 3, p = 0,0021; and block 1 vs 4, p = 0,0001) but not in forwards. When being tackled, SampEn showed significant between-position differences in block 1 (p = 0.0007) and block 3 (p = 0.0118). Significant between-block differences were only observed for backs in block 1 vs 4 (p = 0,0025). Movement variability shows a progressive reduction with cumulative tackle events, especially in backs and when in the defensive role (tackling). Forwards present lower movement variability values in all blocks, particularly in the first block, both in the attacking and defensive role. Entropy measures can be used by practitioners as an alternative tool to analyse the temporal structure of variability of tackle actions and quantify the load of these actions according to playing position.

## INTRODUCTION

Rugby league are physically demanding team sports characterised by a high frequency of tackle events [1]. As such, tackles result in considerable increases in total energy expenditure [2] and upper-body neuromuscular and perceptual fatigue [3]. Furthermore, tackles require high levels of physical fitness and a set of coordinated movement patterns [4]. Consequently, developing tackle and contact abilities becomes an essential aspect of training prescription in the rugby codes [4]. Tackle actions are performed during both defensive and attacking phases of play [5], but defensive tackling may prove crucial in determining the match outcome if they are able to prevent the attacking team progressing towards their try line and scoring a try [6–8]. Therefore, it is important that defensive players are able to maintain the intensity and technique during defensive tackles. Tackle characteristics are different between playing positions, with forwards being involved in more tackles than backs during a match, with the highest frequency recorded for hit-up forwards group (i.e., 35 to 48) compared to the outside backs group (i.e., 23 to 32) [9–11]. Thus, players have different collision-profiles and require different collision training to adequately prepare for the physical-technical characteristics of competitions [12–14].

To adequately prepare for the physical-technical characteristics of competitions, microtechnology is used to study match demands to inform training. Research on the use of microtechnology to quantify external loads in team sports has grown exponentially in the last years [3, 15, 16]. However, most of the research has focused on global positioning system (GPS) derived variables (e.g. distance, high-speed running, accelerations, and decelerations), with limited focus on collisions (e.g., the tackle) [17]. Some micro-technology devices contain multiple components such as accelerometers or gyroscopes, which may provide valuable information related to human movement, with application to the tackle [17]. For instance, accelerometer-derived metrics can be used to quantify the number and magnitude of collision events [12, 15, 16]. In the existing literature, tackle analyses have typically quantified the magnitude of these events and potential changes in technique [4, 6, 12, 18]. Also, the most demanding passages of rugby league match-play involve cumulative tackles and collisions with short recovery between efforts [19]. At the elite level, rugby league players can often be exposed to between 29 and 74 collisions (i.e., tackles and carries) per game [11, 19], or to more than 3 collisions per minute [20]. As such, cumulative tackles events may deteriorate tackle technique and efficiency over time by affecting its movement variability, especially when a player is required to make repeated tackles with their non-dominant side [21, 22], although to date limited research exists in this area.

Movement variability can be defined as a certain amount of change during athletic performance (e.g. a tackle) and perceived as a key element for identifying the amount of perturbation (incidents that change a system state from a stable to an unstable situation or vice versa) in a specific sporting action [23–25]. Therefore, human movement variability can provide an additional tool for quantifying the tackle demands of team sports. Human movement analysis has evolved to assess the variability of a measure by targeting the detection of changes in fluctuations and spatiotemporal characteristics of its outcomes. Linear analyses of human movement have several recognised limitations, mainly in determining the degree of complexity and the time–dependent structure of a time series [26]. These limitations can be complemented by using non-linear analyses, such as measures of entropy. The advantages on this method lie in the additional information on the way in which the levels of a biological system are related, and the organization of athlete’s movement from a dynamical system perspective [27]. Currently, the most commonly used methods for biological data are approximate entropy (ApEn) and, more recently, sample entropy (SampEn) and multiscale entropy (MSE) [27–32]. All of them, are mathematical algorithms to quantify the amount of regularity and the unpredictability of fluctuations over time-series data. However, they can be considered particularly appropriate for the study of sports movements, although it appears that SampEn is more reliable for short data sets [33]. SampEn measures the probability that similar sequences of points in the time-series remain similar within a tolerance level when a point is added to the sequence, in a single time scale [34]. Furthermore, entropy provides researchers the ability to quantify complexity setting high regularity as low entropy and a very random movement as high entropy. Within the past 20 years, entropy analysis has gained popularity in movement sciences in sports to describe changes in postural control [35–38], assessment of running [39, 40], human walking data [41–43], tactical behaviour in soccer [44, 45], force production [46–48] and as a measure of system complexity in sports [49]. Recently, it has also been validated for detecting increases in movement variability in elite rugby players during resistance training when a ball is included [50, 51].

To our knowledge, no study has explored the movement variability in tackle actions and its changes during repeated tackles. Therefore, the aim of this study was to identify between-position (forwards *vs.* backs) differences in movement variability in cumulative tackles events training during both attacking and defensive roles.

## MATERIALS AND METHODS

### Subjects

Eleven elite adolescent male rugby league players (mean ± SD, age; 18.5 ± 0.5 years, height; 179.5 ± 5.0 cm, body mass; 88.3 ± 13.0 kg) were recruited for this study, six forwards and five backs. All participants were selected from a single professional rugby league academy based in England. Prior to volunteering, the experimental protocol was explained to all participants both verbally and in writing, with a written statement of consent signed (in the case of minors, players provided assent and parents provided consent). The procedures complied with the Declaration of Helsinki (2013) and were approved by Leeds Beckett University Research Ethics Committee.

### Design and Methodology

**FIG. 1:**
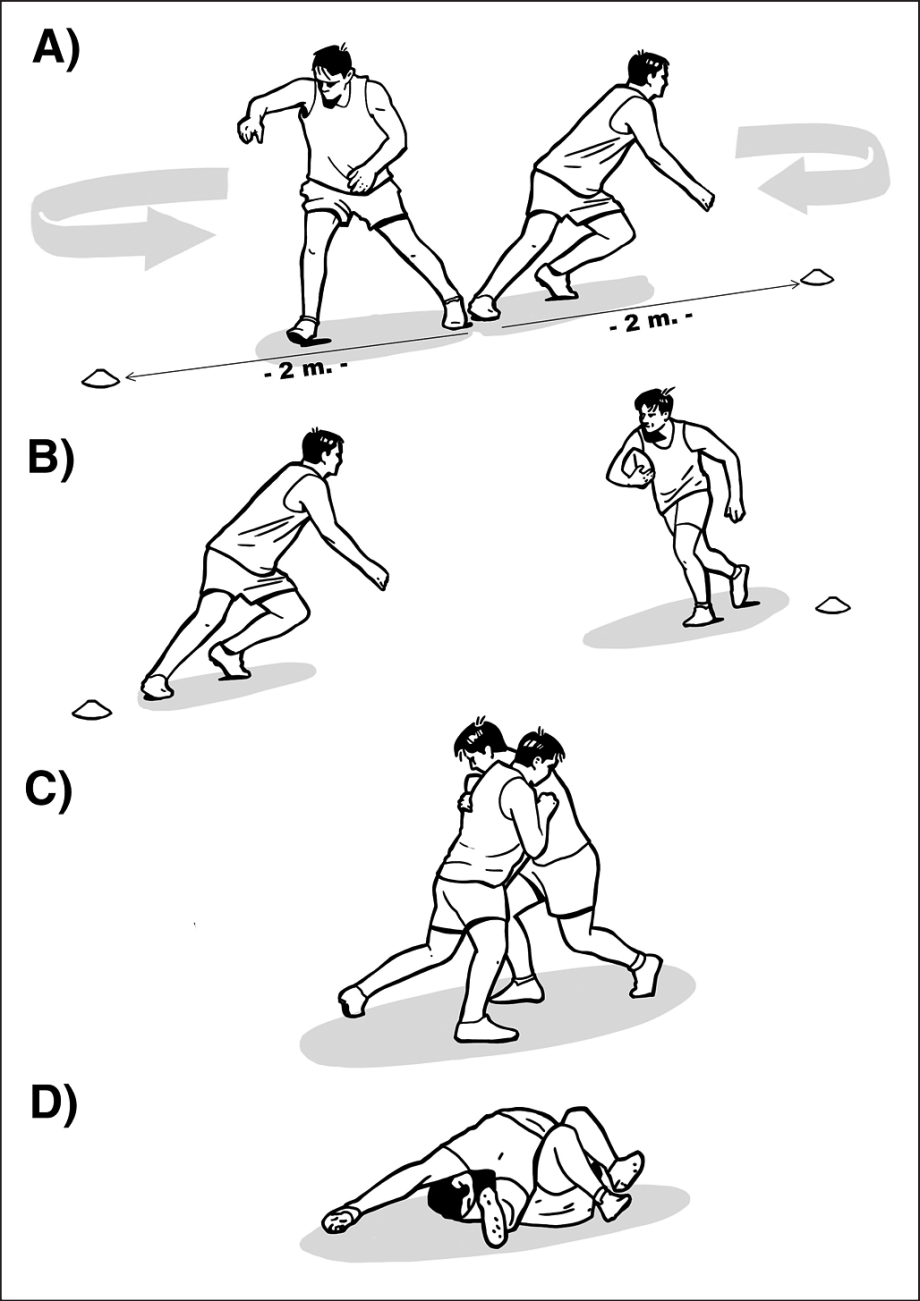
One-on-one tackles. Players started in front of each other, when the coach marked the start the players crossed two meters in the opposite direction (A) and then changed direction (B) to execute the tackle at the central point (C, D).

Participants performed a drill encompassing 48 one-on-one tackles divided into 24 *tackling* (i.e. tackling an opponent) and 24 *tackled* (i.e. being tackled by an opponent while carrying a ball) events. These drills were structured in four blocks, and each block consisted of six *tackling* and six *tackled* activities in random order. The players started in front of each other, when the coach marked the start, the players crossed two meters in the opposite direction and then changed direction to execute the tackle at the central point (Fig. 1). The players were divided by positions (e.g., forwards or backs), so that they were always paired with a player of their same position. The experimental protocol began with a standardised 10-minute warm-up. Participants were instructed and encouraged to tackle with maximum effort. During *tackling* actions, participants alternated between shoulders (i.e. three tackles using the dominant shoulder and three tackles using the non-dominants shoulder) within each block. Ninety seconds of passive recovery was prescribed between each block. Professional coaches directed the sessions to ensure session safety and ecological validity. The prescribed 48 collisions account for more than the match demands reported for professional rugby league [52] and rugby union [53], to induce a greater level of tackle induced fatigue. A total of 528 tackles were analysed (288 for forwards and 240 for backs).

Participants wore a micro-technology inertial measurement unit (WIMU, Realtrack Systems, Almeria, Spain), which was tightly fitted to the athletes upper back with a specialised vest to minimise incidental unit movement and enhance reliability [17]. The micro-technology units contain a 10 Hz Galileo GPS positioning device, a 3D accelerometer; 100G recording at 1000 Hz, a 3D gyroscope recording at 1000 Hz. The devices were calibrated prior to their placement. This was done with a self-calibration system that incorporates each device in the internal configuration of the boot. During self-calibration, three aspects were taken into account: (i) leaving the device immobile for 30 s; (ii) placing it in a flat area; and (iii) no magnetic devices around it [54]. These devices have reported good results in accuracy and reliability of his different sensors in previous studies [54–58].

The raw acceleration signal was extracted from each device (from fig 1B to 1D), and processed using a summation of vectors (AcelT) in three axes, mediolateral (x), anteroposterior (y) and vertical (z) calculated according to Gómez-Carmona et al. (2018) [56]. AcelT indicates only the acceleration, in g-force values, recorded by the 3D accelerometers that make up the inertial device with a sample frequency of 1000 Hz, all without the application of a calculation to modify the raw data from the signal. Therefore, if accelerometers recorded the AcelT variable in a reliable form, all variables calculated using accelerometers would be reliable [56]. To obtain a clean acceleration signal, an optimum filter process related to the different sample frequencies was applied [56].

Two of the most widely used and successful entropy estimators are Approximate Entropy (ApEn) and Sample Entropy (SampEn) [31]. ApEn quantifies the similarity probability of patterns of length m and m + 1. SampEn is a similar statistic, and it also measures the probability of subsequences being close at two lengths m and m + 1. However, SampEn does not include self–comparisons and exhibits greater consistency than ApEn [31]. For this reason, we used SampEn for the current study. Mathematical equation of SampEn is [34, 59]:

1) Form *m*-vectors, *X(1)* to *X(N-m+1)* defined by
  X(i)=[x(i),x(i+1),…,X(i+m−1)] i=1,N−m+1 (1)
2) Define for each i, for i = 1, N-m, let
  $$B_{i}^{m}\left(r\right)=\frac{1}{N-m+1}\times no.ofd_{m}\left[X\left(i\right),X\left(j\right)\right]\leq r,i\neq j$$ (2)
3) Similarly, define for each i, for i = 1, N-m, let
  $$A_{i}^{m}\left(r\right)=\frac{1}{N-m+1}\times no.ofd_{m+1}\left[X\left(i\right),X\left(j\right)\right]\leq r,\;i\neq j$$ (3)
4) After define:
  $$B^{m}\left(r\right)=\frac{1}{N-m}\sum_{i=1}^{N-m}B_{i}^{m}\left(r\right)$$ (4)
  $$A^{m}\left(r\right)=\frac{1}{N-m}\sum_{i=1}^{N-m}A_{i}^{m}\left(r\right)$$ (5)
5) Finally:
  $$SampEn\left(m,r,N\right)=-\text{ln}\left(\frac{A^{m}\left(r\right)}{B^{m}\left(r\right)}\right)$$ (6)

The (AcelT) signal was cut separating each collision, obtaining 48 signals for each subject and device. Sample entropy (SampEn) for each signal were calculated. Entropy was done according to Goldberger et al. [60] and through dedicated routines programmed in Matlab^®^(The MathWorks, Massachusetts, USA). We used the template length m of 2, and the tolerance criterion of 0.20 in the analyses.

### Statistical Analysis

Descriptive analyses are reported as mean ± standard deviations. Data normality and homogeneity was assessed using Shapiro-Wilk and Levene tests, respectively. Data analyses were conducted using PASW Statistics 21 (SPSS, Inc., Chicago, IL, USA). Independent sample T-tests were used to evaluate differences in SampEn between positions in each block, one for attacking and other for defensive roles. Four linear mixed-effects models were used to model the main and interactive effects between blocks for dependent variables (SampEn) divided between-position and attacking and defensive role (forwards attacking, forwards defensive, backs attacking and backs defensive). The ‘ID’ of the player was treated as the fixed effect, whereas the random effect was ‘block’ for all analyses.

The comparisons were also assessed via standardised mean differences (Cohen’s d) and respective 90% confidence intervals. Thresholds for effect sizes statistics were < 0.20, trivial; 0.20–0.59, small; 0.6–1.19, moderate; 1.20–1.99, large; and > 2.0, very large [61]. For all statistical tests, a p < 0.05 was considered statistically significant.

Within-block acceleration variability was analysed using coefficient of variation expressed as a percentage of the mean acceleration signal (CV%) and was represented using box and whisker plots. The box and whisker plots display the first and third quartiles as the ends of the box, the maximum and minimum as the whiskers and the median and average as a vertical bar and + symbol respectively in the interior of each box.

## RESULTS

**FIG. 2:**
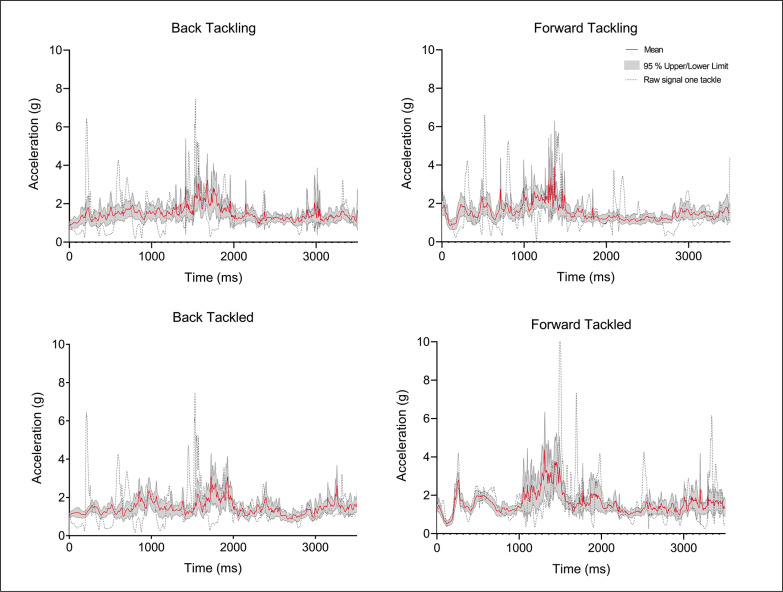
Raw data of acceleration signal of one tackle, mean over blocks and 95% confidence interval for one player in each condition. Back tackling, back tackled, forward tackling and forward tackled.

The average and standard deviation of SampEn values for tackling and tackled for forwards and backs in each block are shown in Table 1. Moreover, raw data (acceleration signal) of one tackle are shown as example in figure 2.

**TABLE 1:** Means (± SD) of Sample Entropy values between-position (forwards *vs*. backs) during both attacking and defensive role.

		Backs	Forwards
Block 1	Tackling	0.085 ± 0.012	0.067 ± 0.013
	Tackled	0.085 ± 0.010	0.069 ± 0.014

Block 2	Tackling	0.079 ± 0.006	0.066 ± 0.008
	Tackled	0.077 ± 0.011	0.073 ± 0.011

Block 3	Tackling	0.072 ± 0.008	0.066 ± 0.007
	Tackled	0.081 ± 0.006	0.069 ± 0.009

Block 4	Tackling	0.069 ± 0.008	0.063 ± 0.006
	Tackled	0.071 ± 0.011	0.065 ± 0.014

### Tackling

**FIG. 3:**
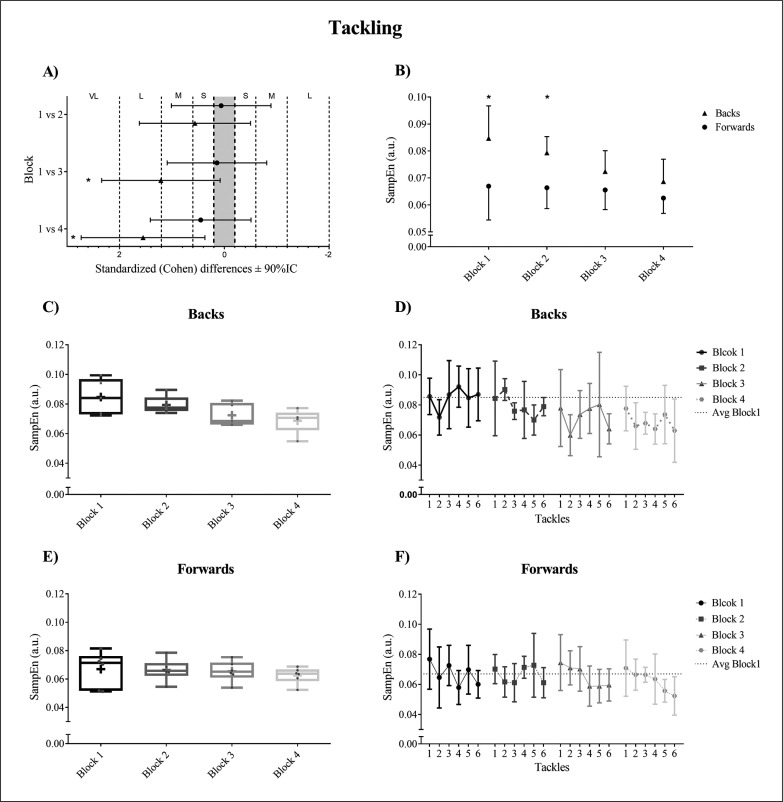
Movement variability when tackling for backs and forwards. (A). Standardised Cohen’s differences between blocks. Error bars indicate uncertainty in true mean changes with 90% confidence intervals. VL: Very Large; L: Large; M: Moderate; S: Small. (B). SampEn (mean ± SD) and T-test analysis between positions within each block. (C, E). Box-and-Whisker-Plots in each block for backs and forwards respectively. (D, F). Average and standard deviation in all defensive tackles for backs and forwards respectively. The significant differences were shown as * p < 0.05.

The duration of tackling in milliseconds (mean ± SD) was 3775.42 ± 312.88 ms for forwards and 3863.47 ± 403.21 ms for backs. Figure 3 shows movement variability when tackling for backs and forwards. Standardised mean differences (Cohen’s d) and linear mixed-effects model with interactive effects between blocks for dependent variables (SampEn) divided into two positional groups (backs and forwards) for *tackling* is shown in Figure 3A. In backs, significant differences were observed in block 1 vs 3 (p = 0.0021) (ES = -0.560) and in block 1 vs 4 (p = 0.0001) (ES = -1.550), but not in block 1 vs 2 (p = 0.2002) (ES = -1.210). No significant differences were observed in any of the studied block comparisons in forwards (block 1 vs 2, p = 0.8290, ES = -0.060; block 1 vs 3, p = 0.6102, ES = -0.140; block 1 vs 4, p = 0.1155, ES = -0.450). Figure 3B shows SampEN (mean ± SD) and T-test analysis for differences between positions within each block. Significant between-position differences were observed in block 1 (p = 0.0001) (ES = 1.44) and block 2 (p = 0.0003) (ES = 1.88) but not in blocks 3 and 4 (p = 0.1664, ES = 0.92 and p = 0.0899, ES = 0.87, respectively). Also, backs showed lower movement variability in all tackles compared with block 1. In contrast, forwards only showed a clear decrease in the last tackles of the last blocks (Fig. 3D, 3F).

Within block variability when tackling was higher for forwards (CV; 18.7% block 1; 11.63% block 2; 11.06% block 3; 9.11% block 4) vs (14.23% block 1; 7.56% block 2; 10.60% block 3; 12.01% block 4) for backs. Also, box and whisker plots showed more variability within blocks for forwards than backs except in the last block (Fig. 3C, 3E).

No differences were observed in dominant vs. non-dominant shoulder neither in forwards (p = 0.067) nor in backs (p = 0.345).

### Tackled

**FIG. 4:**
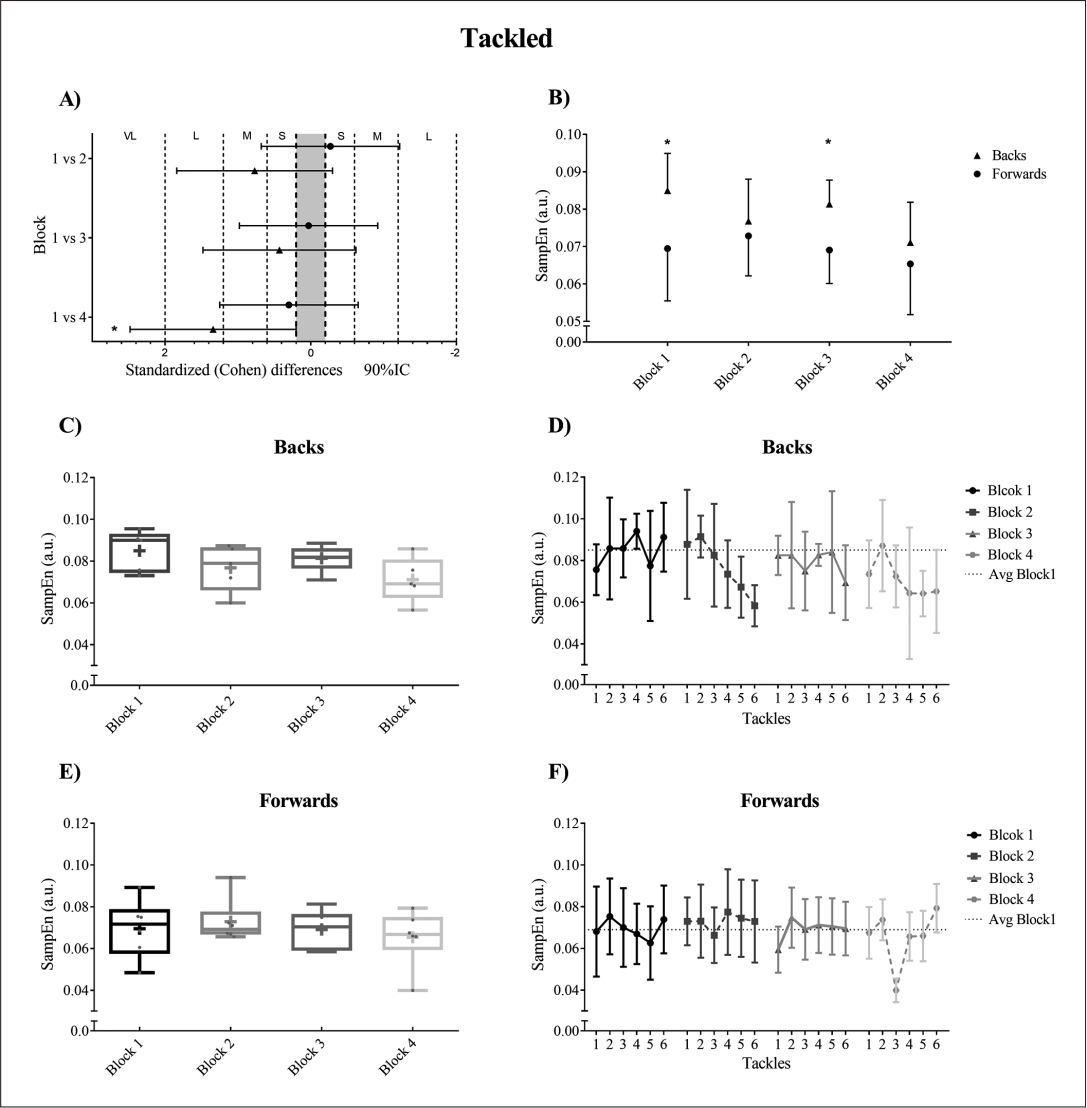
Movement variability when being tackled for backs and forwards. (A). Standardised Cohen’s differences between blocks. Error bars indicate uncertainty in true mean changes with 90% confidence intervals. VL: Very Large; L: Large; M: Moderate; S: Small. (B). SampEn (mean ± SD) and T-test analysis between positions within each block. (C, E). Box and whisker plots in each block for backs and forwards respectively. (D, F). Average and standard deviation in all attacking tackles for backs and forwards respectively. The significant differences were shown as * p < 0.05.

The duration of tackled in milliseconds (mean ± SD) was 3775.02 ± 390.33 ms for forwards and 3970.27 ± 376.07 for backs. Figure 4 shows movement variability when being tackled for backs and forwards. Standardised mean differences (Cohen’s d) and linear mixed-effects model with interactive effects between blocks for dependent variables (SampEn) divided into two positional groups (backs and forwards) for *tackled* are shown in Figure 4A. In backs, significant differences were observed in block 1 vs 4 (p = 0.0025) (ES = -1.340), but not in block 1 vs 2 (p = 0.0756) (ES = -0.770) or block 1 vs 3 (p = 0.2321) (ES = -0.430). No significant differences were observed in any of the studied block comparisons in forwards (block 1 vs 2, p = 0.2779, ES = 0.270; block 1 vs 3, p = 0.8949, ES = -0.030; block 1 vs 4, p = 0.1830, ES = -0.300). Figure 4B shows SampEN (mean ± SD) and T-test analysis for differences between positions within each block. Significant between-position differences were observed in block 1 (p = 0.0007) (ES = 1.28) and block 3 (p = 0.0118) (ES = 1.56) but not in blocks 2 and 4 (p = 0.3939, ES = 0.36 and p = 0.2132, ES = 0.47, respectively). Also, backs showed higher movement variability in all tackles compared with block 1, in contrast forwards only showed a clearly decrease in the last tackles of the last block (Fig. 4D, 4F).

Within block variability when being tackled showed higher levels of variability for forwards (CV; 20.16% block 1; 14.63% block 2; 12.97% block 3; 20.72% block 4) than for backs (11.71% block 1; 14.61% block 2; 7.90% block 3; 15.14% block 4). Also, box and whisker plots show a higher within-block variability in forwards than in backs except in block 2 (Fig. 4C, 4E).

No differences were observed in dominant vs. non-dominant shoulder neither in forwards (p = 0.482) nor in backs (p = 0.695).

## DISCUSSION

This study aimed to identify changes in movement variability between positions (forwards *vs.* backs) in cumulative tackles events training during both attacking and defensive roles in rugby league. To our knowledge, this is the first study to analyse movement variability in tackling actions. The main findings are that movement variability is progressively reduced with cumulative tackle events over blocks (i.e. six *tackling* and six *tackled*), especially for backs and defensive tackles. Overall, forwards present the lower movement variability in all blocks than backs. Previous research suggests that movement variability might be reduced by different factors; on the one hand as a function of practice or experience [51, 62], and on the other hand because of aging [27], disease [27], injury [63] or fatigue [64]. Therefore, since forwards perform more collisions during the course of a match [12] this might suggest that forwards adjust better to tackle actions.

In the current study, it appears that forwards maintain their levels of movement variability without significant changes during cumulative tackle events. In contrast, backs present higher levels of movement variability in block 1 and suffer significant decreases with cumulative tackle events. In such a manner, when the interactions among elements in the system worsen, the movement variability could be reduced affecting locomotor outputs [27]. Gabbett and Ryan (2009) [18] found that the greatest improvements in tackling technique occurred in the players with the lowest initial technical tackling ability. This behaviour has also been found in the present study, since the players who presented higher initials levels of movement variability showed the greatest decreases in movement variability. Thus, the between-position differences observed in movement variability initial values and its behaviour during cumulative tackle events are probably associated with specific positional requirements [12].

Cummins & Orr (2015) [12] showed that both forwards and backs experienced more collision events in defence than attack. Consequently, the principal focus of the tackle task should be set on defensive tackles because these may prove crucial in determining match outcomes [6]. Running speed is progressively reduced when players (especially backs) are required to perform a high number of collisions per minute [20]. The present study shows a similar behaviour in movement variability, especially in tackling actions. The major difference was produced between block 1 and 4 with a small decrease in movement variability in forwards and large changes with significant differences in backs (Fig. 3A). This highlights that the decrease in movement variability in backs is due to the fact that the majority of the tackles are below the average of the entropy values of block 1 (Fig. 3D). However, and in forwards only, the last tackles in blocks 3 and 4 are below the average of block 1 (Fig. 3F). Also, a clear association between the decrease in movement variability and an increase in the number of contact effort in defensive actions exists (Fig. 3B). In this sense, if movement variability is low it might harm a player’s tackling ability, and in turn potentially increase the risk of injury [63], so a change in the structure of the task could be suggested. The attacking play of hit ups and the ability to tolerate physical collisions is important for rugby league players [12]. Similar to tackling actions, when participants were being tackled both positional groups showed a progressive reduction in movement variability. However, the last block was only significantly different in backs (Fig. 4A).

Understanding the tackle characteristics and quantifying its load should be an essential part of load monitoring in rugby and could be associated with tackle performance [4]. Current tackle analysis using microtechnology is limited to counting the number of tackle events and their magnitude. Until now, the most widely used tool is an algorithm designed specifically for rugby league, which quantifies collision counts [65]. This algorithm is sensitive to detect 97.6% of collision events during professional rugby league match-play [65]. However, Glazier & Davids (2009) [66] state that it is the structure, rather than the magnitude, of variability is important in uncovering the functionality of this ubiquitous feature of human motor behaviour. Moreover, Wu et al. (2014) [67] suggested that the temporal structure of motor output variability can explain differences in how individuals adapt to different types of dynamics. The differences found between backs and forwards in entropy calculated from accelerometers in our study reflects the different forms of adaptation to the environment derived from the specificity of the training by positions in the same team and probably should be taken into account to plan the training. Thus, entropy could be a good alternative tool to analyse the temporal structure of variability in tackle actions and to understand the differences in locomotor outputs between position when performing multiple collisions training.

Futures studies should focus on analysing changes in movement variability during open tasks with decision-making components and during match play and if it is associated with match-play tackling performance. Furthermore, commercially available accelerometers usually sample at a frequency of 100 Hz [17], so the validity of tri-axial accelerometers sampling at 100 Hz for calculating entropy in short actions like a tackles frequencies should be assessed to fully understand if this analysis can be extended to other commercially available devices.

### Limitations

The current study was performed on a single professional rugby league academy squad and during a single standardised training session with controlled tackling and tackled movements. While the number of tackles analysed was 528, more than the match demands reported for professional rugby league [52], the tackle was performed in a controlled setting and may not fully represent real match conditions. Studying the tackle in controlled settings however, allows for experiential and explorative study designs, which offers deeper insight into the demands and patterns of the movement [68]. Also, when considering the findings between positions, it is worth noting that tackle event occurred within the same position i.e. forwards competed against forwards, and back competed against backs. As such, how tackle variability may behave when a forward competes against a back (on attack and defence) is not known, and a potential avenue for future research. Also, the participants were elite adolescent male rugby league players, so the findings of this study can be useful for researchers and practitioners working at the elite level.

## CONCLUSIONS

To our knowledge, this is the first study to use entropy analysis to quantify the changes in movement variability in cumulative tackle events in elite rugby players. In conclusion, movement variability is progressively reduced with cumulative tackle events, especially in backs and in the defensive role. Forwards present lower movement variability values in all blocks, particularly in the first block, both in the attacking and defensive role.

### Practical Implications

1. Entropy measures can be used by practitioners as an alternative tool to analyse the temporal structure of variability of tackle actions and to quantify the load of these actions by positions.
2. Movement variability analysis can help to maintain the optimal complexity in repetitive tackle tasks between positions.
3. Practitioners should modify the contact tasks between positions to adjust the complexity of the task to the different requirements of each position and difference collisions-profiles to optimize the training process.
